# Specific interventions as part of a quality improvement collaborative enhance care for ischemic stroke patients

**DOI:** 10.3389/fneur.2026.1830076

**Published:** 2026-10-06

**Authors:** Charlotte Lens, Ellen Coeckelberghs, Deborah Seys, Kris Vanhaecht, Kim Bekelaar, Roel Blondia, Tom Bogaert, Laura Boutens, Joke Caudron, Romina Demontis, Sylvie De Raedt, Laurens Dobbels, Anneke Govaerts, Dimitri Hemelsoet, Marijke Huskens, Petra Olislaegers, Jean Francois Poma, Leen Sinnaeve, Charlotte Swinnen, Annelies Terwecoren, Peter Vanacker, Noa Vandevenne, Wendy Van Daele, Jens Van Elsuë, Jan Vynckier, Laetitia Yperzeele, Jelle Demeestere, Caroline Weltens, Robin Lemmens

**Affiliations:** 1 Department of Public Health, Leuven Institute for Healthcare Policy, KU Leuven—University of Leuven, Leuven, Belgium; 2 Department of Neurosciences, Experimental Neurology, KU Leuven—University of Leuven, Leuven, Belgium; 3 Department of Quality Management, University Hospitals Leuven, Leuven, Belgium; 4 Department of Neurology, Ziekenhuis Oost Limburg, Genk, Belgium; 5 Department of Neurology, AZ Alma, Eeklo, Belgium; 6 Department of IT and Care, Jan Yperman Hospital, Ypres, Belgium; 7 Department of Quality Management, AZ Glorieux, Ronse, Belgium; 8 Department of Neurology, AZ Sint Blasius, Dendermonde, Belgium; 9 Department of Neurology, Jessa Hospital, Hasselt, Belgium; 10 Department of Neurology, University Hospital Brussel (UZ Brussel), Brussels, Belgium; 11 NEUR Research Group, Vrije Universiteit Brussel (VUB), Brussels, Belgium; 12 Department of Neurology, Imelda Hospital, Bonheiden, Belgium; 13 Department of Neurology, University Hospitals Leuven, Leuven, Belgium; 14 Department of Neurology, RZ Heilig Hart, Tienen, Belgium; 15 Department of Neurology, Ghent University Hospital, Ghent, Belgium; 16 Department of Neurology, AZ Maria Middelares, Ghent, Belgium; 17 Department of Nursing and Paramedics, AZ Sint Maarten, Mechelen, Belgium; 18 Department of Neurology, Kliniek Sint Jan, Brussels, Belgium; 19 Department of Neurology, AZ Sint-Lucas, Bruges, Belgium; 20 Department of Neurology, AZ Mol, Mol, Belgium; 21 Department of Neurology, AZ Oostend, Ostend, Belgium; 22 Department of Neurology, AZ Groeninge, Kortrijk, Belgium; 23 Neurovascular Center and Stroke Unit, Department of Neurology, Antwerp University Hospital, Antwerp (Edegem), Belgium; 24 Research Group on Translational Neurosciences, Faculty of Medicine and Health Sciences, University of Antwerp, Antwerp, Belgium; 25 Department of Quality Management, Sint-Franciscus Ziekenhuis, Heusden-Zolder, Belgium; 26 Department of Neurology, ZAS Middelheim, Antwerp, Belgium; 27 Department of Nursing and Paramedics, AZ Sint Elisabeth, Zottegem, Belgium; 28 Department of Neurology, Onze-Lieve-Vrouwziekenhuis, Aalst, Belgium; 29 Department of Radiation Oncology, University Hospitals Leuven, Leuven, Belgium

**Keywords:** adherence, care pathways, guidelines, ischemic stroke, quality improvement collaborative

## Abstract

**Introduction:**

Adherence to guidelines for stroke care varies, despite evidence that managing fever, glycemia (sugar), and conducting swallowing screening (FeSS) reduces mortality and disability. We established a quality improvement collaborative to improve care processes for ischemic stroke, with a focus on these three recommendations. We then added two additional care process activities: screening for activities of daily living (ADL) and depression, collectively defined as FeSS^+^.

**Methods:**

In this multicenter intervention study (*n* = 24), we retrospectively analyzed 30 consecutive patient records per center of ischemic stroke patients in 2019, pre-intervention (measurement period 1 [MP1]), and in 2022, post-intervention (measurement period 2 [MP2]). All hospitals received feedback on their MP1 performance, and an optional e-learning tool on FeSS^+^ was also offered. We compared adherence between MP1 and MP2 and assessed the impact of the e-learning tool.

**Results:**

Data were collected from 580 patients during MP1 and 588 patients during MP2. E-learning tools were delivered to teams at 18 hospitals (75%). Adherence improved for temperature measurement (18 to 25%, *p* = 0.004) and swallowing screening (36 to 59%, *p* < 0.001). No significant changes were observed for glycemia, ADL, or depression screening. Improvements in adherence to glycemia management, swallowing screening, and depression screening were observed only among hospitals that disseminated the e-learning tool (*p* = 0.01, <0.001, <0.001, respectively). Temperature measurement improved more in hospitals without e-learning (OR 2.62 vs. 1.27; *p* = 0.03).

**Discussion:**

We observed improvements in measuring temperature and swallowing screening after receiving feedback. The dissemination of the e-learning tool was associated with increased adherence to glycemic measurement, swallowing screening, and depression screening, underscoring the role of specific interventions. However, overall adherence remained low, highlighting the need for continued quality improvement.

## 1 Introduction

Stroke remains the second leading cause of death and a major contributor to disability worldwide. Evidence has been collected on several key interventions in the stroke care pathway to improve the outcomes of ischemic stroke patients. An important component in achieving this goal is developing evidence-based stroke protocols in the hospital setting (1–4). Despite robust evidence for many interventions, their implementation into clinical practice is not consistently achieved, and variation in the performance of stroke care processes exists (1, 5–7).

International studies, in particular the Quality in Acute Stroke Care (QASC) trials, have demonstrated the beneficial effect of developing and implementing a multidisciplinary, evidence-based protocol for the management of temperature (fever), glycemia (sugar), and swallowing function screening (FeSS protocol) on the outcomes of ischemic stroke patients (2, 7, 8). However, our previous study showed relatively low adherence rates to elements of the FeSS protocol in a cohort of 29 Belgian hospitals (6). The recommendations and guidelines have changed, incorporating additional elements such as screening of activities of daily living (ADL) and depression. We determined limited adherence to these screenings, potentially resulting from the scarcity of guidelines on the prevention, assessment, and treatment of post-stroke depression and ADL screening (9–11).

Quality improvement collaboratives (QICs) are a proven method to improve adherence to guidelines and to implement evidence into daily practice. A QIC is a multifaceted, organized approach that includes teams from multiple healthcare sites. Teams learn, apply, and share ideas, improvement methods, and data on performance for a given healthcare topic. In a QIC, the whole multidisciplinary team is involved in progressing the care process according to the latest evidence (12). To disseminate information to the multidisciplinary teams in the hospitals, e-learning tools can serve as an ideal and practical tool, as they facilitate the distribution of standardized information (13).

To further enhance care for patients with ischemic stroke, we targeted improvements in adherence to the FeSS recommendations, as well as screening for ADL and depression, which we defined as FeSS^+^ recommendations (Table 1). To improve knowledge of these recommendations, an e-learning tool was developed and distributed to hospitals. The aim was twofold: (1) to improve adherence to all five FeSS^+^ recommendations and (2) to evaluate the role of the e-learning tool in achieving this.

**Table 1:** FeSS^+^ clinical treatment protocol for the first 72 h after hospital admission: key elements.

FeSS+ indicator of evidence	Level of evidence
*Fever*	LOE B (2, 9)
Temperature monitored every 4 h after hospital admission for the first 72 h.	
*Sugar (hyperglycemia)*	LOE B-NR (2, 9)
Blood glucose measurement every 1–6 h after hospital admission for the first 72 h.	
*Swallowing*	LOE A (2, 9)
Patients were screened within 24 h of admission to the stroke unit.	
*ADL screening*	LOE B-NR (9)
Performance of ADL screening in eligible stroke patients with a structured screening tool.	
*Depression screening*	LOE B-NR (9)
Performance of depression screening in eligible stroke patients with a structured screening tool.	

Definitions of levels of evidence (LOE) applied in this study: Level A means high-quality evidence from more than one randomized controlled trial (RCT), meta-analyses of high-quality RCTs, or one or more RCTs corroborated by high-quality registry studies; Level B-randomized means moderate-quality evidence from one or more RCTs or meta-analyses of moderate-quality RCTs; Level B non-randomized means moderate-quality evidence from one or more well-designed, well-executed nonrandomized studies, observational or registry studies, or meta-analyses of such studies (9). FeSS^+^, management of temperature, glycemia, swallowing function screening, activities of daily living, and depression screening; ADL, activities of daily living; LOE, level of evidence.

## 2 Methods

### 2.1 Study population

This multicenter intervention study was performed in 24 hospitals in Flanders, Belgium, all participating in a QIC aimed at improving ischemic stroke care. This was a follow-up to the previously reported pre-intervention study, and the inclusion and exclusion criteria were basically similar (6). The participating hospitals were a mix of academic/teaching and non-teaching hospitals, as well as small and large volume hospitals. Patients were included if they were (I) aged ≥18 years and (II) diagnosed with ischemic stroke after admission to the emergency department. Patients were excluded if they were (I) treated with mechanical thrombectomy (transfer of these patients would result in stroke care interventions in two hospitals, hampering a hospital-based analysis) or (II) had an in-hospital stroke (typically, this would not involve admission to the emergency department). From the eligible patient population, we excluded patients with a length of stay of less than 72 h and those who died or had a registered decision for comfort care in the first 72 h, as these patients would typically not follow the stroke care pathway evaluated by FeSS^+^. Ethical approval was obtained from the Ethics Committee of the University Hospitals Leuven and, if needed, from the local ethics committee of the participating centers.

### 2.2 Variables

Table 1 provides an overview of the FeSS^+^ recommendations explored in this study, together with their level of evidence. The definitions and adherence criteria described in Table 1 were applied consistently throughout the data collection and analysis. For indicators requiring repeated measurements, adherence was defined as completion of the full recommended protocol.

### 2.3 Measurement periods and intervention

Two measurement periods (MPs) were analyzed in this study. For the first MP, data were collected in spring 2021. To avoid any influence of the COVID-19 pandemic, pre-intervention data were collected from the last 30 consecutive patients discharged before 31 December 2019 (6). For the second MP, in spring 2023, data were collected from the last 30 consecutive patients discharged before 31 December 2022. Between the two MPs, an intervention and improvement period took place (Figure 1). For each MP, patient data were collected by a local study coordinator using a structured audit tool to analyze patient records.

**Figure 1:**
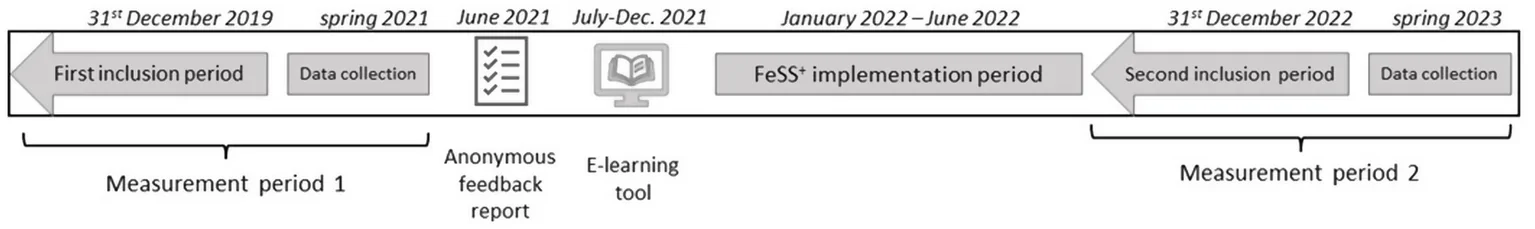
Study overview. Study overview; FeSS^+^, management of temperature, glycemia, swallowing function screening, activities of daily living screening, and depression screening.

The intervention consisted of two components. First, an anonymized feedback report, which provided a comprehensive overview of clinical performance throughout the whole care process from admission to the emergency department until discharge, was prepared. This report, based on the results from MP1, was distributed to all participating hospitals. The second component of the intervention was an e-learning tool, consisting of online educational modules for each of the five FeSS^+^ key interventions. Each module included a brief video in which evidence-based recommendations and their importance were explained. Hospitals had the flexibility to select and utilize any number of these modules.

Which educational modules were used at the hospital level was evaluated through interviews with local study coordinators and by incorporating a related question into the structured audit tool of MP2. Both information sources were used to determine which of the FeSS^+^ educational modules had been distributed to the local teams (Supplementary Table 1). In case of ambiguity, the local study coordinator was contacted via email to clarify whether they had used educational modules and which ones.

### 2.4 Statistical analysis

Patient characteristics were summarized using descriptive statistics and presented as mean ± SD, median (interquartile range [IQR]) or *n* (%). The Kruskal–Wallis test was used to determine differences between the samples of the MP1 and MP2 samples. Adherence rates were calculated as the number of patients for whom the guidelines were adhered to and divided by the number of patients for whom the intervention was indicated. In the structured audit tool, it was possible to indicate if no information was found in the patient record regarding a specific intervention. This was analyzed similarly to “not performed.” Additionally, adherence rates were compared between patients from hospitals that used the educational module for a specific FeSS^+^ recommendation and those from hospitals that did not use the educational module. To evaluate these differences in adherence, a logistic regression analysis with a random intercept for hospitals was used. This analysis accounted for two factors: MP 1 vs. 2 and the use of the educational module. The interaction effect was evaluated to understand the potential modifying role of the educational module on adherence rates.

The primary objective of this study was to improve adherence to the FeSS^+^ recommendations as an overall measure of stroke care quality. Individual FeSS^+^ recommendations were analyzed separately to explore changes in adherence patterns and differences according to the implementation strategy. Because the study was designed as a quality improvement evaluation, the analyses of individual FeSS^+^ indicators were considered exploratory. No adjustment for multiple comparisons was performed.

All analyses used a significance level of *p* < 0.05 and were performed using SAS 9.4.

## 3 Results

### 3.1 Study sample

We received documentation of 1,440 patient records from 24 hospitals, equally distributed across both measurement periods. After applying all inclusion and exclusion criteria, 1,168 patients were eligible for analysis—580 from MP1 and 588 from MP2. Baseline patient characteristics for each measurement period are summarized in Table 2. We noticed an increase in National Institutes of Health Stroke Scale (NIHSS) documentation at admission (59% vs. 76%, *p* < 0.001), as well as in the documentation of hypertension and hypercholesterolemia (both *p* < 0.001). Slightly more patients received treatment with intravenous thrombolysis (20% vs. 21%; *p* = 0.04).

**Table 2:** Overview of the patient characteristics across both measurement periods.

Variable	MP1 (*n* = 580) Mean ± SD, median (IQR) or *n* (%)	MP2 (*n* = 588) Mean ± SD, median (IQR) or *n* (%)
Age (mean ± SD)	72.9 ± 14	74.4 ± 13.6
Sex (*n* (%))		
Male	307 (52.9%)	303 (51.5%)
Female	273 (47.1%)	285 (48.5%)
BMI (mean ± SD)	26.4 ± 4.9	26.1 ± 4.9
NIHSS known (*n* (%))	344 (59.3%)	444 (75.5%)
NIHSS value (median (IQR))	3 (5)	3 (5)
Smoking status (*n* (%))		
Smoker	116 (20%)	115 (19.6%)
Non/former smoker	340 (58.6%)	372 (63.3%)
No information	124 (21.4%)	101 (17.2%)
Diabetes (*n* (%))		
Yes	141 (24.3%)	160 (27.2%)
No	362 (62.4%)	352 (59.9%)
No information	77 (13.3%)	76 (12.9%)
Hypertension (*n* (%))		
Yes	336 (57.9%)	369 (67.4%)
No	164 (28.3%)	156 (26.5%)
No information	80 (13.8%)	36 (6.1%)
Hypercholesterolemia (*n* (%))		
Yes	263 (45.3%)	365 (62.1%)
No	205 (35.3%)	158 (26.9%)
No information	112 (19.3%)	65 (11.1%)
Treated with tPA (*n* (%))	115 (20%)	126 (21%)

BMI, body mass Index; NIHSS, National Institutes of Health Stroke Scale; tPA, tissue Plasminogen Activator; SD, standard deviation; *n*, number.

### 3.2 Comparison of FeSS^+^ adherence rates between the two measurement periods

When comparing adherence rates for the FeSS^+^ recommendations, an increase in temperature measurement from 18% (*n* = 105) to 25% (*n* = 148) (*p* = 0.004) and swallowing function screening from 36% (*n* = 211) to 59% (*n* = 349) (*p* < 0.001) was observed.

Glycemia measurement did not change between the two periods, with rates of 28% (*n* = 164) and 30% (*n* = 178), respectively. Similar findings were observed for ADL screening, 50% (*n* = 292) and 51% (*n* = 298), respectively, and depression screening, which remained at 20% for both MP1 (*n* = 116) and MP2 (*n* = 118). Odds ratios (ORs) and 95%CIs are shown in Figure 2.

**Figure 2:**
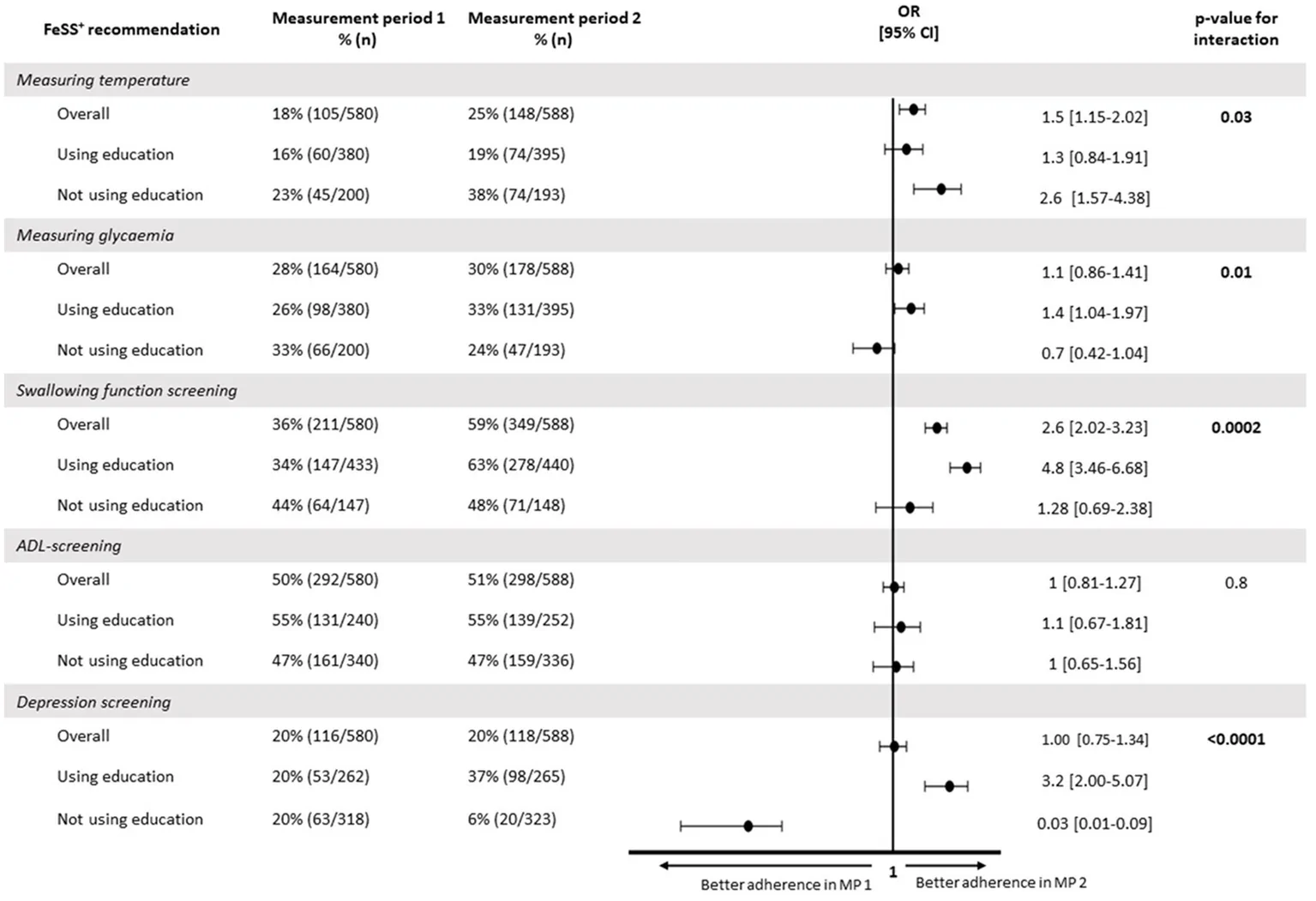
Overview of adherence rates across measurement periods 1 and 2 and differences related to the educational module use. Overall indicates a comparison of the adherence rates between measurement period 1 (*n* = 580) and measurement period 2 (*n* = 588). Hospitals had the option to disseminate the educational module for a specific FeSS^+^ recommendation. As module dissemination was not randomized, hospitals that reported using the module were compared with hospitals that did not report using the module, and the results should be interpreted as associations rather than causal effects. Odds ratios (ORs) were used to compare adherence in hospitals that used the module with that in hospitals that did not. The *p*-values for the interaction effect are shown in the last column. α = 0.05. “Education” refers to the educational module that was disseminated. MP, measurement period.

### 3.3 Comparison of FeSS^+^ adherence rates taking the e-learning tool into account

All hospitals were part of the QIC and received the feedback report and an additional e-learning tool containing educational modules on each FeSS^+^ recommendation. Distribution of the tool within each hospital was at the discretion of the team leads. We collected information on the dissemination of these modules to study the role of this intervention in adherence to the recommendations. Overall, 18 of the 24 hospitals (75%) used at least one educational module from the e-learning tool. Educational modules were used in 16 hospitals (67%) for temperature and glycemia, 18 (75%) for swallowing function screening, 10 (42%) for ADL screening, and 11 (46%) for depression screening (Supplementary Table 1).

For three FeSS^+^ recommendations, improved adherence rates were observed in hospitals where these tools had been disseminated. These were glycemia measurement, from 26% (98 of 380) to 33% (131 of 395) (p for interaction 0.01); swallowing function screening, from 34% (147 of 433) to 63% (278 of 440) (p for interaction 0.0002); and depression screening, from 20% (53 of 262) to 37% (98 of 265) (p for interaction <0.0001). Interestingly, temperature adherence improved more in hospitals without the e-learning tool (23 to 38%) than in those with the e-learning tool (16 to 19%; p for interaction 0.03). ORs and 95% CIs are shown in Figure 2.

## 4 Discussion

This study aimed to improve key components of the care process for patients with ischemic stroke within a QIC by assessing adherence to the five FeSS^+^ recommendations in 1,168 patients (580 pre-intervention and 588 post-intervention) across 24 hospitals (6). By providing a feedback report on performance across the entire care process, adherence rates to temperature measurement and swallowing function screening improved overall. Adherence to glycemia measurement, swallowing function screening, and depression screening improved in hospitals that used e-learning tools, including educational modules, in addition to the information from the feedback report.

This multicenter intervention study was conducted in the context of a QIC, a widely used method for promoting evidence-based practices in healthcare. QICs are effective as they facilitate the sharing of data and innovations between hospitals. Teams tend to learn more quickly and implement improvement ideas more effectively when they collaborate and benchmark with others, as was initiated in this study by the feedback report (12, 14, 15). Typically, teams in a QIC are supported by a group of experts, who identify and inform on best practices. We increased our efforts toward participating hospitals beyond the feedback report by developing e-learning modules per recommendation (12, 14–17). E-learning is generally feasible and valuable for quality improvement (13, 18). Healthcare professionals in stroke care prefer mini modules on key clinical topics (19). The e-learning module developed for this QIC met many of the requirements known to increase its effectiveness and feasibility: The content was directly applicable on the medical ward; the time investment to peruse the module was limited; and it contained information on the rationale and scientific background for each quality improvement recommendation (13, 20). Our findings suggest that the educational modules may reinforce adherence to at least three FeSS^+^ recommendations beyond the effect of feedback alone. However, it should be noted that the observed differences between hospitals that disseminated e-learning modules and those that did not should be interpreted cautiously. Dissemination of the educational modules was based on local team decisions and was not randomly assigned. Therefore, hospitals adopting the modules may have differed in unmeasured characteristics, such as organizational motivation, leadership engagement, quality improvement culture, or available resources. These factors may have contributed to both the decision to disseminate the modules and the observed improvements in FeSS^+^ adherence.

In the overall cohort, we observed improvements in temperature measurements according to the FeSS protocol. However, the overall adherence rate of only 25% post-intervention remains low. Interestingly, contrary to the other FeSS^+^ indicators, the greatest improvement in temperature adherence was observed in hospitals that received feedback only rather than those that additionally disseminated the e-learning module. Temperature was measured in all patients, but not as thoroughly as required by the FeSS protocol. We received feedback that medical teams considered the measurements too frequent over the 72 h period in patients without any clinical signs of fever in the acute phase. An adaptive protocol depending on the findings during the first 24–36 h could be explored for both efficacy and feasibility on stroke units. Similar considerations may apply to other FeSS parameters, such as glycemia. Although regular monitoring is recommended in the acute phase of stroke, rigid monitoring schedules may be challenging to maintain in clinically stable patients and may increase the workload for nursing teams. Future implementation strategies should therefore explore adaptive, risk-based monitoring approaches that preserve patient safety while improving feasibility and sustainability in routine clinical practice.

For this particular indicator, distribution of the e-learning tool was not associated with improved adherence compared with using the feedback report alone. This finding is of interest because it suggests that the added value of educational interventions may depend on the complexity and characteristics of the targeted care process. Several factors may have contributed: First, the e-learning intervention was embedded within the broader context of a QIC, which can already have beneficial effects on care processes (12, 14, 16, 21–23). All hospitals were informed about guideline-recommended stroke care, including temperature management, which is relatively easy to implement, enabling an increased performance over the two measurement periods. Second, some of these hospitals may have prioritized improving temperature measurements through local initiatives. Unfortunately, we did not collect information about these actions beyond the QIC. Third, for temperature management, a researcher presented the module video, while in the other modules, stroke care professionals delivered the content. Healthcare professionals may respond better to guidance from peers than to guidance from researchers (24). Feedback alone may be sufficient for simple, visible, and measurable key interventions in the care processes, whereas educational interventions may provide more added value for complex key interventions requiring knowledge translation or behavioral change.

The greatest improvement in adherence from MP1 to MP2 was observed for swallowing function screening. This was more pronounced in hospitals that provided the educational module to their stroke teams compared with those that only used the feedback report. Similar findings were also noted in the original QASC trial in Australia and its European translation (2, 7). During discussions on MP1 with the participating hospitals, it became apparent that swallowing function screening differed between hospitals. In the educational module, we outlined the important role of a trained nurse. This resulted in clear, easy-to-follow instructions. Additionally, the direct link between screening and the prevention of pneumonia was outlined. Understanding the critical role of the intervention and the associated responsibilities in the care pathway likely strengthened the motivation and need to adequately screen for dysphagia.

Relatively low adherence rates for measuring glycemia align with previous Flemish data (6, 25). A possible explanation is that lower nurse staffing in Belgian hospitals compared with other countries, combined with time constraints, may hinder the delivery of complete care (26). This concern was also identified in the FeSS protocol pre-implementation study, where insufficient nursing staffing was identified as a barrier (27). Despite this challenge, glycemia monitoring remains crucial, as hyperglycemia is common in ischemic stroke and is associated with larger infarct size and poorer outcomes (28). Specific interventions may increase awareness and thereby improve practice, as shown in our results. Hospitals that disseminated the educational module in addition to the information they received via the feedback report showed greater progress when comparing the two measurement periods. Repeated sessions may help to maintain and enhance the performance of stroke teams.

Despite one-third of patients experiencing post-stroke depression, which is directly associated with suicide, morbidity, and poor recovery, depression screening rates remained low (11, 29–31). We did observe an effect of the e-learning tool, which supports the role of stroke team education when implementing new elements in stroke care. Previous research identified barriers to post-stroke depression screening, including a lack of screening tools, increased workload, and insufficient motivation and knowledge (30). Strategies such as support from clinical champions, the use of accessible screening tools, and educational programs, as implemented in this study, can help overcome these challenges (30).

The proportion of ADL assessments remained low and nearly unchanged between MP1 and MP2. Occupational therapists, who typically conduct this assessment and provide therapeutic interventions, reported several barriers such as poor communication, cognitive impairment, low patient educational levels, and language barriers. Similar to nursing staff, occupational therapists face difficulties in completing all required tasks, a challenge worsened by fluctuating staffing levels (32). Nonetheless, we believe that evaluating the effect of stroke on ADL is essential for appropriate discharge and rehabilitation planning.

Adherence rates for both ADL and depression screening remained low; this can also be interpreted in light of the strength of the underlying guideline recommendations. In contrast to swallowing screening, which is supported by Level A evidence, recommendations for ADL assessment and depression screening are based on Level B-NR evidence. In the context of limited time and staffing resources, healthcare professionals may understandably prioritize the implementation of recommendations supported by the strongest evidence and perceived as having the greatest immediate impact on patient safety. Although this hypothesis was not formally evaluated in this study, differences in the strength of the supporting evidence may have influenced local implementation priorities and should be considered when interpreting the observed adherence patterns. Future quality improvement initiatives may benefit from tailoring implementation strategies according to both the complexity of the care process and the strength of the supporting evidence.

Although we included 1,168 patients in this multicenter intervention study across 24 hospitals, we acknowledge several limitations. First, the study used an uncontrolled pre-post design without a separate control group. Consequently, improvements observed between the baseline measurement in 2019 and the follow-up measurement in 2022 cannot be attributed exclusively to the quality improvement collaborative, as other time-related factors, including secular trends, healthcare system recovery following the COVID-19 pandemic, and national policy developments, may also have contributed. Although all participating hospitals operated within the same healthcare system and geographical region and were therefore exposed to broadly similar external influences, the absence of a control group means that the independent contribution of these external factors cannot be fully distinguished from that of the intervention. Nevertheless, the observed improvements are consistent with an effect of the quality improvement collaborative. This limitation is inherent to many large-scale quality improvement collaboratives, because the primary objective is to improve care across participating organizations rather than to withhold the intervention from a subset of hospitals. The study was designed and conducted according to the IHI Breakthrough Series Collaborative methodology, a well-established and widely applied approach for large-scale quality improvement initiatives. Nevertheless, the observed improvements are consistent with an effect of the quality improvement collaborative. Second, all participating hospitals were informed about the content of the e-learning tool and its recommendations, and all received the feedback report. Therefore, even if some hospitals reported not using certain FeSS^+^ educational modules, they were never completely isolated from the intervention. Third, the implementation of quality improvement recommendations was left at the discretion of local treatment teams. Consequently, we could not objectively document the extent of implementation and information dissemination. Fourth, all participating hospitals were located in Flanders and voluntarily participated in the QIC. Therefore, caution is needed when extrapolating the findings to healthcare systems with different organizational structures, resources, or implementation contexts. However, FeSS^+^ implementation has previously been evaluated in other settings, including Australia and several European countries. This suggests that the underlying care recommendations have relevance beyond the Flemish healthcare context, although local adaptation remains important (1, 2, 7). Finally, the QIC was established in the aftermath of the COVID-19 pandemic, which was a challenging time for the healthcare sector. Many hospitals encountered difficulties with staffing and resources, which may have hindered the implementation of quality improvement efforts (33).

## 5 Conclusion

Overall, improvements in adherence rates were observed between MP1 (pre-intervention) and MP2 (post-intervention) for temperature measurement and swallowing function screening. The additional e-learning tool may have modified hospital performance, since we documented improved performance for glycemia measurement, swallowing function screening, and depression screening only in hospitals that used e-learning modules. These findings underscore the role of specific interventions beyond participation in an overall QIC. However, adherence rates remain relatively low, highlighting the need for awareness, continued efforts, and better strategies to further enhance the quality of stroke care.

## Data Availability

The raw data supporting the conclusions of this article will be made available by the authors, upon reasonable request.
