# Supplementary material for: Specific interventions as part of a quality improvement collaborative enhance care for ischemic stroke patients
Source: Front Neurol. 2026 Oct 6;17:1830076. doi: 10.3389/fneur.2026.1830076 (PMC13680235; doi:10.3389/fneur.2026.1830076)
Supplement: Supplementary file 1 [file Table_1.docx]

Supplementary Material

# 1 Supplementary Table

Table 1: Overview of the implementation of the 5 FeSS^+^ key interventions at the hospital level

|  | Temperature measurement | Glyceamia measurement | Swallowing function screening | ADL-screening | Depression screening |
| --- | --- | --- | --- | --- | --- |
| 1 | 0 | 0 | 0 | 0 | 0 |
| 2 | 1 | 1 | 1 | 1 | 1 |
| 3 | 1 | 1 | 1 | 0 | 1 |
| 4 | 1 | 1 | 1 | 1 | 1 |
| 5 | 1 | 1 | 1 | 0 | 0 |
| 6 | 1 | 1 | 1 | 1 | 1 |
| 7 | 1 | 1 | 1 | 0 | 0 |
| 8 | 0 | 0 | 1 | 0 | 1 |
| 9 | 0 | 0 | 1 | 0 | 0 |
| 10 | 1 | 1 | 1 | 1 | 1 |
| 11 | 0 | 0 | 0 | 0 | 0 |
| 12 | 1 | 1 | 1 | 1 | 0 |
| 13 | 1 | 1 | 1 | 1 | 0 |
| 14 | 1 | 1 | 1 | 1 | 0 |
| 15 | 1 | 1 | 1 | 0 | 0 |
| 16 | 0 | 0 | 0 | 0 | 0 |
| 17 | 1 | 1 | 1 | 1 | 1 |
| 18 | 1 | 1 | 1 | 1 | 1 |
| 19 | 0 | 0 | 0 | 0 | 0 |
| 20 | 1 | 1 | 1 | 0 | 1 |
| 21 | 1 | 1 | 1 | 1 | 1 |
| 22 | 1 | 1 | 1 | 0 | 1 |
| 23 | 0 | 0 | 0 | 0 | 0 |
| 24 | 0 | 0 | 0 | 0 | 0 |

For each hospital and each recommendation 1 indicates that the hospital used the educational material to implement the recommendations for that specific key intervention.
